# Understanding the prion-like behavior of mutant p53 proteins in triple-negative breast cancer pathogenesis: The current therapeutic strategies and future directions

**DOI:** 10.1016/j.heliyon.2024.e26260

**Published:** 2024-02-10

**Authors:** Yasaman Naeimzadeh, Amir Tajbakhsh, Jafar Fallahi

**Affiliations:** a Department of Molecular Medicine, School of Advanced Medical Sciences and Technologies, Shiraz University of Medical Sciences, Shiraz, 7133654361, Iran; b Pharmaceutical Sciences Research Center, Shiraz University of Medical Sciences, Shiraz, Iran

**Keywords:** Prion-like aggregation, Triple-negative breast cancer, Mutant p53, Cancer treatment

## Abstract

Breast cancer (BC) is viewed as a significant public health issue and is the primary cause of cancer-related deaths among women worldwide. Triple-negative breast cancer (TNBC) is a particularly aggressive subtype that predominantly affects young premenopausal women. The tumor suppressor p53 playsa vital role in the cellular response to DNA damage, and its loss or mutations are commonly present in many cancers, including BC. Recent evidence suggests that mutant p53 proteins can aggregate and form prion-like structures, which may contribute to the pathogenesis of different types of malignancies, such as BC. This review provides an overview of BC molecular subtypes, the epidemiology of TNBC, and the role of p53 in BC development. We also discuss the potential implications of prion-like aggregation in BC and highlight future research directions. Moreover, a comprehensive analysis of the current therapeutic approaches targeting p53 aggregates in BC treatment is presented. Strategies including small molecules, chaperone inhibitors, immunotherapy, CRISPR-Cas9, and siRNA are discussed, along with their potential benefits and drawbacks. The use of these approaches to inhibit p53 aggregation and degradation represents a promising target for cancer therapy. Future investigations into the efficacy of these approaches against various p53 mutations or binding to non-p53 proteins should be conducted to develop more effective and personalized therapies for BC treatment.

## Highlights

• Mutant p53 forms prion-like structures in breast cancer, especially TNBC.
• Targeting p53 aggregation is key in TNBC therapy.
• Diverse approaches (e.g., chaperone inhibitors, aggrephagy-based approaches, immunotherapy, gene therapy, and CRISPR-Cas9) target p53 aggregates.
• Zinc metallochaperones (e.g., ZMC1) and small molecules (e.g., PRIMA-1 and APR-246) show promise in restoring mutant p53 function.

## Graphical abstract

The role of Prion-like Behavior of Mutant p53 Proteins in Triple-Negative Breast Cancer Pathogenesis.

**Figure:**
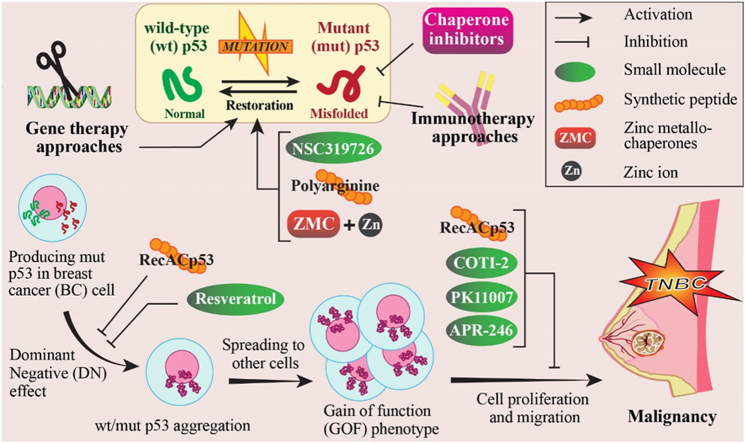

## Abbreviations list

**ANV**: Albumin nano-vector
**ATF6**: Activating transcription factor 6
**BC**: Breast cancer
**BD**: Basic domain
**BRCA1**: Breast cancer gene 1
**BBC3**: BCL2 binding component 3
**C-T**: Carboxylic-terminus
**CCT**: Cytosolic group II chaperonin
**CDKN1A**: Cyclin-dependent kinase inhibitor 1A
**CRISPR**: Clustered regularly interspaced short palindromic repeats
**DBD**: DNA binding domain
**DN**: Dominant negative;
**DNAJA1**: DnaJ Heat Shock Protein Family
**DO-1**: Decrease colocalization
**ER**: Estrogen receptor
**ERAD**: ER-associated degradation
**GOF**: Gain of function
**HCC**: Hepatocellular carcinoma
**HER2**: Human epidermal growth factor receptor-2
**HSP**: Heat shock protein
**IRE-1**: Inositol-requiring enzyme-1
**IHC**: Immunohistochemistry
**LOF**: Loss of function
**MD**: Molecular dynamic
**MDM2**: Mouse double minute 2
**MHC**: major histocompatibility complex
**NDP52**: Nuclear dot protein 52
**OD**: Oligomerization domain
**PARP**: Poly (ADP-ribose) polymerase
**PCD**: Programmed cell death
**PEITC**: Phenethyl isothiocyanate
**PERK**: Protein kinase RNA-like ER kinase
**PR**: Progesterone receptor
**PRD**: Proline-rich domain
**RNase**: Ribonucleases
**ROS**: Reactive oxygen species
**TAD**: Transactivation domain
**TCR**: T cell receptor
**TCRm**: T cell receptor mimic
**TD**: Tetramerization domain
**TAX1BP1**: TAX1 BINDING PROTEIN 1
**TNBC**: Triple-negative Breast Cancer
**UPR**: Unfolded-protein response
**ZMCs**: Zinc metallochaperones
**ZnCl**: Zinc chloride.

## 1 Introduction

Breast cancer (BC) is the second most common cause of death from cancer and the most prevalent cancer in females worldwide [[1], [2], [3]]. A new definition of BC molecular subtypes based on immunohistochemical staining was issued *via* St Gallen International BC Conference 2013: human epidermal growth factor receptor-2 (HER2), Ki67^+^ < 20%), luminal A (estrogen receptor (ER)^+^/progesterone (PR)^+^, luminal B (ER^+^/PR^+^ < 20%, HER2, Ki67^+^ ≥ 20%), HER2 overexpression (ER^−^, PR^−^, HER2^+^), HER2^+^ B2 (ER/PR^+^, HER2^+^), and basal-like TNBC (ER^−^, PR^−^, HER2^−^) [4]. Epidemiological data demonstrate that Triple-negative breast cancer (TNBC) occurs mostly in young premenopausal women (i.e., under 40 years old), who constitute nearly 15–20% of all BC cases. Approximately half of the patients with this BC subtype show distant metastasis with a high recurrence rate after surgery, short survival time after metastasis, and a high mortality rate [5].

Tumor suppressor P53 is a zinc ion-contained homotetramer transcription factor, known as “the guardian of the genome”. It has been observed that more than 50% of cancer patients have various mutations in the *TP53* gene, and it is the most mutated gene in malignancies [[6], [7], [8], [9], [10]]. It plays a key role in different cellular functions, such as growth arrest, DNA repair, apoptosis, and cell senescence [6,11,12]. Generally, the lack of expression of p53 or mutated p53 in human cancers is associated with enhanced tumor growth and resistance to treatment [7]. Indeed, despite the cellular mechanisms of protein folding control, through dominant negative (DN) mutation in *TP53* gene under abnormal conditions, mutant misfolded p53 proteins can be produced and aggregate with each other and with wild-type (wt) p53 that ultimately can lead to ER stress and form pathological prion-like aggregations (e.g., amyloid fibrils) with gain of function (GOF) phenotype and without tumor-suppressor activity that can act as cancer stimulators [11,[13], [14], [15], [16], [17]].

Amyloid aggregations of mutated p53 have been observed in several of BC tissues compared with normal tissue [6,12,18]. However, although TNBC is highly heterogeneous, mutant p53 has been found in approximately 80% of these cases [19,20]. Thus, such a high prevalence makes targeting mutant p53 one of the main research priorities for treating TNBC cases that do not have an effective targeted therapy. In this review, structure and function of p53, the process of aggregation of abnormal p53 in a cell that causes endoplasmic reticulum (ER) stress, and the role of prion-like p53 structures in cancer development, especially TNBC will be discussed. Ultimately, different therapeutic approaches based on p53-mutated targeted therapy for BC patients will be discussed.

## 2 Impairment of protein hemostasis results in ER stress

Approximately one-third of cellular proteins are produced in the ER, which is responsible for processing, folding, trafficking, and maintaining protein homeostasis *via* synthesis, degradation, and repair of proteins [12,21,22]. These molecules should gain appropriate folding and dynamics to warrant their proper activities and avoid cellular stress that can eventually lead to malignant transformation. Pending evolution, cells gained specialized mechanisms to regulate folding, post-translational changes, quality and maturation of synthesized proteins [12]. Moreover, chaperones ([heat shock protein (Hsp)70 and Hsp90]] contribute in structural maturation and target misfolded proteins for ubiquitination and proteolysis, whereas chaperonins aid in the appropriate folding of new generated or misfolded proteins within their structural context [23]. Furthermore, key amino acid interactions are necessary for proper protein conformation [12].

**Fig. 1:**
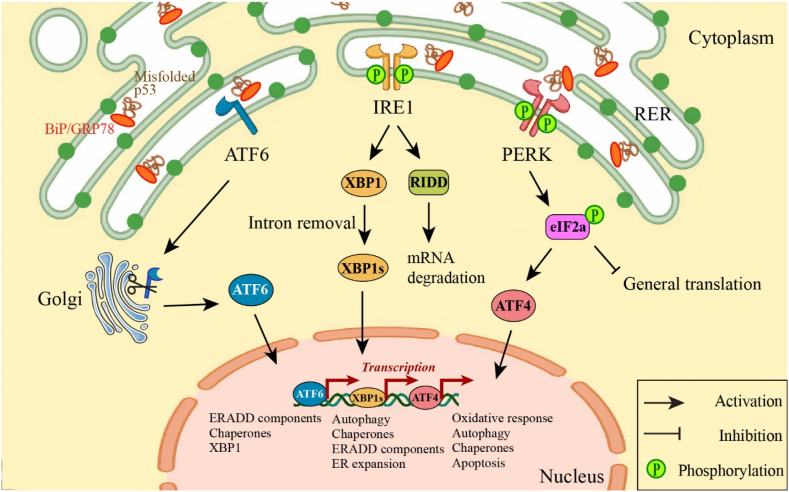
Upon misfolded protein accumulation or ER stress, GRP78/BiP, an ER chaperone, permits IRE1, PERK, and ATF6 (stress sensors) above to activate downstream signaling. IRE1 activation mediates the generation of spliced XBP1 mRNA, which is translocated to the nucleus to stimulate UPR target genes. The RNase domain of IRE1 also regulates the RIDD pathway, where IRE1 degrades ER membrane-localized mRNAs by its RNase activity, which leads to a decline of imported protein into the ER lumen. Also, PERK activation can induce the phosphorylation of the alpha subunit of the eIF2 (a translation protein), which declines protein translation to decrease ER protein overload, while upregulating ATF4 mRNA, which can stimulate apoptosis. Also, ATF6, is translocated to the Golgi apparatus, for cleavage. The cleaved fragments are then translocated to the nucleus and activate the transcriptional target genes of ATF6. **ATF6**: Activating transcription factor 6; **eIF2**: Eukaryotic initiation factor 2; **ER**: Endoplasmic reticulum; **GRP78/BiP**: Binding Immunoglobulin Protein; **IRE1**: Inositol-requiring enzyme 1; **PERK**: Protein kinase RNA-like ER kinase; **RIDD**: Regulated IRE1-dependent decay; **UPR**: Unfolded protein response; **XBP1**: X-box binding protein 1.

Cells can experience the ER stress through the protein folding deficiency and protein overload within the ER (Fig. 1). Following this phenomenon, cells can activate the “unfolded-protein response” (UPR) [21,23,24] that triggers 3 signaling sensors, including protein kinase RNA-like ER kinase (PERK), inositol-requiring protein-1α (IRE-1α), and activating transcription factor 6 (ATF6) [25]. UPR helps the cell to eliminate ER stress by reducing mRNA transcription and protein translation and increasing chaperones to eliminate the concentration of misfolded proteins in ER lumen. ER-associated degradation (ERAD) pathways [23,26], including the ubiquitin/proteasome pathway (ERAD I) and the autophagic/lysosomal pathway (ERAD II) are the other mechanisms for remaining misfolded proteins, which have escaped from UPR process. If these processes do not work, programmed cell death (PCD) pathways will be activated, involving apoptosis (PCD1), autophagy (PCD2), or necrosis (PCD3) [12].

## 3 Structure and function of p53

Tumor suppressors p53 can function in both transcription-dependent (direct) and transcription-independent (indirect) [[27], [28], [29]]. It plays the role of a transcription factor *via* binding to special DNA sequences [e.g., mouse double minute 2 (MDM2), Cyclin-dependent kinase inhibitor 1A (CDKN1A), BAX, and BCL2 binding component 3 (BBC3)], using DNA binding domain (DBD), and exerting its anti-proliferative effects in cell. However, in some cases, it can interact with other proteins and indirectly control the target gene [27,[29], [30], [31]]. In fact, under normal physiological conditions, the p53 half-life in the cell is not too long, and it is degraded by cellular proteins, including MDM2. For example, MDM2, which has ubiquitin E3 ligation activity, connects to the N-terminal p53 and leads to proteasome degradation [11]. However, under genotoxic stresses, p53 is activated *via* post-translational modifications, including phosphorylation, acetylation, and methylation. Activated p53 can connect to the promoter of downstream target genes and increase the expression level of target genes [6].

The human *TP53* gene encodes a p53 homotetramer protein containing 393 amino acids, which are highly evolutionarily conserved [6,12]. On the basis of structural analysis, it has been observed that the full-length p53 contains five functional domains: 1) Transactivation domain (TAD) at the amino-terminus (N-T), 2) Proline-rich domain (PRD), 3) DBD which is also named core domain, 4) tetramerization domain (TD) [or oligomerization domain (OD)], and 5) Basic domain (BD) (or regulatory domain) at the carboxylic-terminus (C-T) [6,29]. TAD is crucial for p53 stability, where p53 binds to its negative regulator (i.e., MDM2) and ultimately suppresses p53 transcriptional activity. PRD is an essential region for p53-dependent apoptosis and transcriptional activity [29,32]. Moreover, the lack of this domain enhances the affinity of MDM2 for p53 and leads to degradation; thus, it is considered an important regulatory domain for p53 [11]. The central DBD directly binds to the DNA binding site of p53 [29] and has a length of approximately 92–94 amino acids and includes a central scaffold-like immunoglobulin β-sandwich [6,33,34]. The other structural units of this domain together form the surface of DNA binding that includes a loop-plate-helix motif and two large loops [34]. The oligomeric state of p53 can be adjusted using TD. In addition, it has been shown that sequence-specific binding of the central DBD is regulated by BD interaction with nonspecific DNA sequences *in vitro* [29].

P53 is a transcription factor containing 6 zinc ions. These ions bind to the amino acids Cys176, Cys242, Cys238, and His179, which are essential for stabilizing and maintaining the structure of the second and third rings of DBD and ultimately lead to proper p53 function [6,7,35,36]. In addition, the formation of a p53 hemotetramer by oligomerization domain increases p53 transcriptional activity [6].

## 4 Types of p53 mutations

P53 is mainly affected by missense mutations or non-synonymous single-nucleotide variants, which usually occur in the core domain that generates the full-length mutant p53 proteins [6,8,11,37,38]. However, synonymous, non-synonymous, frameshift, silent, graft mutations, and mutations affecting post-translational mutations in *TP53* have been reported in various types of tumors as well [11]. It has been demonstrated that missense mutations, which lead to amino acid replacement in the p53, are involved in the pathogenesis and weak prognosis of more than half of malignant tumors. Furthermore, several studies were indicated that the formation of mutated p53 aggregations is related to the loss of function (LOF), dominant-negative (DN), and GOF mutations at the hot spots of the amino acid sequence of this protein [13,37,39]. Although complete LOF mutations are counted as the important hallmarks of p53 mutations that influence cancer response to chemotherapy, GOF mutations in this protein are observed in some cancers that have various effects, including enhanced migration, invasion, and metastasis [6,9,11,40,41]. According to crystal structure studies on p53 protein, its missense mutations are divided into two main categories; the first category contains p53 contact mutations (e.g.*, R248Q, R248W*, *R273H*, and *R273C*) in which the mutated protein sustains the wt protein conformation, but it carries a mutation in the DBD that impresses its proper binding to DNA. The second category includes conformational (structural) mutations (e.g., *R175H*, *Y220C*, *R249S*, and *R282W*) that affect the conformation and overall structure of this protein [6,7,11,13,42,43]. The last group can reduce p53 melting temperature, which forms a misfolded state at physiological temperature [35,42,44]. Moreover, several mutations in coordinator amino acids, including *C176F*, *H179R*, *C238S*, *C242S*, *R273H*, and *R175H* lead to the loss of zinc ions (Apo-p53) and consequently decreased p53 affinity to DNA binding and structural instability [7,35,45]. In fact, mutant p53 is prone to loss of zinc ions from the DBD domain, leading to inhibition of tetramer formation, protein misfolding, and aggregation [7,11,46]. It has been revealed that loss of zinc ions, due to the *Y220C* mutation in *p53*, could expose the hydrophobic cavities or amyloidogenic region (residues 251–257), which is terminated by p53 aggregation [47].

Structural mutations in DBD, as the most prevalent mutations in *p53*, can affect its function due to inappropriate protein folding, which dramatically reduces the potency of p53 to bind to its target genes [6,9,[48], [49], [50]] and could lead to the aggregation of misfolded p53 that can take many forms, including amyloid oligomers and fibrils, and promote oncogenic functions [13,51]. Misfolded P53 containing contact mutations is not essential because DBD usually exhibits correct folding. However, these two extensive categories of contact and structural missense mutations in p53 may overlap with each other, and they do not always act in a specific and unique way; for example, some contact mutations, such as *R248Q* can also have structural consequences [6,11,44]. The cytosolic group II chaperonin (CCT) has been identified as a part of the p53 interactome. In fact, proper folding of wt p53 requires interaction with CCT, and even in the absence of normal mutations in DBD, the lack of interaction with this chaperone can lead to oncogenic activities of p53 [12].

Several scenarios can justify how mutated p53 can participate in malignancy: 1) conformational changes in DBD; 2) Changes in the interactome of p53 (e.g., transcription factors, and accessory proteins) that can cause it to bind to other cellular partners; and 3) different types of aggregated species and formation of various oligomeric, amyloid, and amorphous structures [52].

## 5 Misfolded p53 protein and cancer

Despite the above mechanisms of protein folding control, misfolded protein production is not always an unerring process and can have various destructive effects on the cells. Currently, approximately 40 misfolded protein diseases, including neurodegenerative diseases, obesity, diabetes, prion diseases, and cancers have been found, which result in improper folding of proteins and their aggregation in cells [12,23,[53], [54], [55], [56], [57]] (Table 1).

**Table 1:** Misfolded protein diseases and their targets.

Name of Disease	Target Protein	Name of Disease	Target Protein
AD	Aβ [181], tau [182]	CJD	PrP [183]
HD	Huntingtin [184]	PD	α-synuclein [185]
Sickel cell anemia	HbS [186]	RP	Rhodopsin [187]
Type II diabetes	Amylin [188]	Fabry	α-galactosidase [189]
Cystic fibrosis	CFTR [190]	Desminopathy	Desmin & α β-crystallin
SBMA	AR [191]	EBS	Keratin 5 [192], Keratin 14 [193]
TTR-FAP	TTR [194]	ALS	SOD1 [195]
Spinocerebellar ataxia	Ataxin [196]	NDI	Aquaporin2/vasopressin
DRPLA	Atrophin-1 [197]	Multiple myeloma	Antibody light chain
Hypogonadotropic hypogonadism	GNRH [198]	A1ATD	AAT [199]
NPCD	NPC1 [200]	Hypertrophic & dilated cardiomyopathy	MYH7, MYBPC3 & TTN
Cancer	P53 [14]	Gaucher's disease	β-glucosidase [201]
Cancer	SRC [202]	Familial insomnia	PrP [203]

**Abbreviations list: A1ATD**: α1-Antitrypsin deficiency; **AD**: Alzheimer's disease; **ALS**: Amyotrophic Lateral Sclerosis; **Aβ**: Amyloidβ; **CFTR:** Cystic fibrosis transmembrane conductance regulator; **CJD**: Creutzfeldt-Jakob disease; **DRPLA**: Dentatorubropallido-Luysian atrophy; **EBS**: Epidermolysisbullosa simplex; **HbS**: Hemoglobin S; **HD**: Huntington's disease; **NDI**: Nephrogenic diabetes insipidus; **NPCD**: Niemann Pick Type C disease; **PD**: Parkinson's disease; **RP**: Retinitis pignemtosa; **SBMA**: Spinal bulbar muscular atrophy; **SRC:** Steroid Receptor Coactivator; **TTR-FAP**: Transthyretin familial amyloid Polyneuropathy.

In various cancers, different metabolic and transcriptional abnormalities can generate adversary microenvironments by impairing ER homeostasis in tumor cells. These changes can promote a state of continuous ER stress that can control multiple pro-tumoral attributes in malignant cells [21].

In cancer, cells have genetic flexibility and adaptability to survive in harmful environments. However, cancer cells adapt by stimulating angiogenesis, in the process of growing solid tumors, the rate of angiogenesis is not in equilibrium with high cell proliferation; in fact, the required nutrients and oxygen are more powerful than those surrounded by the vascular network [12]. This imbalanced situation causes hypoxia, oxidative stress, decreased pH (lactic acidosis), and decreased glucose and amino acid reserves [21]. Hypoxia, as an electron carrier, disrupts the formation of disulfide bonds, and decreased glucose levels influence protein glycosylation and ATP production. All of these factors contribute to incorrect proteins accumulation within the ER lumen and stimulate UPR. Unlike normal cells, which under ER stress stimulate cell survival or death through UPR signals, cancer cells continue to grow under adverse conditions by escaping UPR-induced death signals. For instance, a gradual decrease in pH, p53 tends to form a melted spherical composition to adapt to the aforementioned environment [12,58]. Mutated p53 can be linked to various types of cancer by evading ER quality control mechanisms and creating a DN effect on its wt counterpart [12].

## 6 Concept of amyloid, prion, and prion diseases

The phrase "amyloid" is generally used for all proteins that are able to form large, insoluble fibers [[59], [60], [61]]. Although amyloid fibers perform biological functions, they are usually associated with pathology. “Prion” is a type of amyloid fibril that can to convert a natural endogenous cellular protein into an amyloid compound and distribute these fibrils between the cells. In fact, the prion assumption was first proven when prions were transferred to yeast cells in the form of pure amyloid fibers, causing the transmission of prion infection over several generations [62,63]. In prion degenerative diseases, properly folded proteins converts to their misfolded versions, resulting in GOF of that protein, ultimately leading to cell death. Accordingly, prion diseases are considered disorders that share many common features with degenerative neurological diseases [11,12,64].

## 7 The origin story and concept of p53 prion-like in cancer

### 7.1 The origin story of p53 prion-like behavior in cancer

The term “prion p53” was first mentioned in 1995 [65]. Research indicated thatmutant p53 proteins (carrying p. R151S, p. R247I, p. R273P, p. R273L) are able to change the wt p53 with normal conformation to the mutant conformation when co-translated, which was considered as a basis explanation of DN effect of mutant P53 [66,67]. Moreover, it has been observed that in baker's yeast with co-expressed wt and mutant p53, the transcriptional activities of wt p53 were suppressed through the DN effect of mutant p53, and as soon as the expression of mutant p53 was blocked, wt p53 recovered its transcriptional activities completely [68]. Though yeasts lack endogenous p53, human p53 expression in them is considered as a powerful assay for evaluation of the transcription factor function of human p53 [69,70]. Such a study demonstrated that the DN effect of mutant p53 is dose-dependent and can be decreased or neutralized *via* wt/mutant p53 ratio enhancement [67]. Furthermore, transient upregulation of prion proteins in yeast can stimulate the proteins to form aggregates that are self-seeding [71,72]; so, transient upregulation of mutant or wt p53 can enhance the chance of p53 inactivation, de novo prion formation, aggregation and aggregate replication. It can be a possible explanation for finding inactive wt p53 is in the cytoplasm of several tumor cells, including BC cells [[73], [74], [75]]. As well as, in current research, it has been demonstrated the transient upregulation of p53 could stimulate the generation of p53 prion aggregates which were transmitted more than 100 generations in yeasts [76]. The transcription factor activity of p53 was declined *via* prion formation, proposing that prion aggregation could lead to cancer [76].

On the other hand, the conformational flexibility of p53 has prepared the foundation for the p53 prion hypothesis, which has been investigated through their reactivity to conformation-specific antibodies; for example, pAb1620 antibody could recognize the wt p53 conformation, whereas pAb240 antibody recognizes the mutant p53 conformation [77,78]. Indeed, the clear potential of some p53 mutations to promote a conformational exchange to the wt p53 conformation, along with the availability of alternate p53 conformations, established the "prion p53" theory [79]. Several immunohistochemistry (IHC) investigations have also shown that mutant p53 proteins can create protein aggregates that improperly accumulate in the nucleus and cytoplasm of several types of tumor sample cells [[80], [81], [82], [83]]. Despite significant differences in the quantity and characteristics of p53 aggregation (e.g., depending on p53 mutation and cell type), the International Society of Amyloidosis nomenclature committee defined p53 as the genuine amyloid [67,84,85].

### 7.2 Concept of prion-like p53 in cancer

Lately, various studies have extended the concept of prion to the pathological accumulation of misfolded p53 proteins in malignant tumors. Although all mutant p53 domains can form amyloid granules, the DBD has the greatest tendency to form amyloid oligomers and fibers [11,13,51]. The basis of the DN mutation in cancer is that mutant p53 proteins accumulate in ER by escaping from quality control mechanisms and convert wt p53 proteins to the aggregated species, which can be transferred to other cells by penetrating the cell membrane. These events lead to an oncogenic GOF phenotype and tumor suppressor LOF and eventually can lead to malignancy [11,[86], [87], [88], [89]]. In other words, the wt and mutant p53 are concurrently expressed in the cells of patients who are in the early stage cancer cells with a somatic mutation in one TP53 allele or carry the germline mutation; therefore, heterotetramers with wt and mutant p53 proteins are formed, which are able to induce prion properties of aggregated p53, and these heterotetramers are unable to bind to its genetic targets [29,90,91]. The GOF functions of aggregated p53 could be applied to the cell by influencing the expression of genes associated with oncogenic properties, direct binding to new DNA regulatory elements, binding to other cytosolic or nuclear factors, or *via* other non-transcriptional functions of aggregated p53 proteins [52].

Several studies have revealed in addition to the aggregation of mutant p53 with its wt: this molecule can also form prion-like aggregations with its paralog tumor suppressors, such as p63 and p73 [11,13,36,43,91]. Bioinformatic studies have shown that there is a conserved susceptible peptide (amino acids 251–258) in the DBD of wt p53 protein that accumulates and forms fibrils from this region and can aggregate with mutated p53, p63, and p73 and exhibit prion-like behavior [11,92]. In fact, molecular dynamic (MD) simulations have shown that these amino acids are prone to aggregation [11]. In addition, it has been demonstrated that p53 aggregation could terminate with the overexpression of Hsp70, as an anti-apoptotic factor [92].

## 8 Prion-like p53 and breast cancer

In 65–80% of biopsy samples of TNBCs, as the most invasive BCs, the p53 is mutated. In fact, current investigations have indicated p53 is mutated in 12% of luminal type A, 29% of luminal type B, 72% of HER2-enriched (HER2^+^), and 80% of basal-like breast tumors [6,11,12]. Several studies have shown that most cases of BC gene 1 (*BRCA1*) BC, as one of the most common types of BC, are associated with the p53 mutation that indicates a potential link between *BRCA1* and *p53* [93,94]. Moreover, according to documented studies, mutated p53 of amyloid oligomers in MDA-MB-231 (commonly serves as a model for TNBC) tumor cells is more common versus in wt p53 (i.e., MCF7) cells, which indicates the prion-like and oncogenic role of mutant p53 in malignant BCs [11,12]. However, aggregated wt p53 was indicated in a 3D model of MCF-7 BC cells, and 5-fluorouracil (5-FU) therapy could further enhance wt p53 aggregation, partial chemoresistance, and suppression of apoptosis [95].

Specific mutations of *p53*, such as *R175H*, *H193L*, *I195L*, *Y234C*, *G245S*, or *R248Q*, have been observed in the biopsies of patients with BC [11,93,94] that involve structural mutations in p53. Therefore, BC may be due to the prion-like behavior of the p53 mutant, which is due to its GOF mutations.

## 9 Cancer treatment using protein targeting methods

Inhibition of p53 aggregation, which is the most frequent mutant protein in various cancers, seems to be an engaged goal for the remedy of patients with malignant tumors; however, p53 aggregation is also a sophisticated phenomenon, and each mutant has a different behavior [[96], [97], [98]]. Thus, several approaches are available for targeting both wt and mutant p53 to disrupt the formation of aggregation and fibrils for cancer treatment [17,36,[99], [100], [101], [102]]. In addition to inhibition of forming p53 aggregates, some strategies including gene therapy are also focusing on activating mutant p53 and its hyper-activation in different types of cancer, including BCs.

### 9.1 Ion treatment

Findings highlight the role of zinc ion in reactivating the function of the mutant p53; therefore, by maintaining zinc ion concentration and re-establishing binding of this ion to mutant p53, it is feasible to restore the function of wt p53 by binding to DNA and activating its related target genes, which can lead to tumor growth inhibition in response to the drug [103,104]. For instance, it has been observed that zinc ion supplements by zinc chloride (ZnCl_2_) can help to suppress the oncogenicity of p53, restore its wt binding to target promoters, and activate apoptotic transcriptional activity in response to the drug by reactivating H175 and H273, which can have better results for the treatment of cancer patients [[105], [106], [107]]. Reducing the interaction between p53 and p73 and restoring the binding of both p53 and p73 to their target gene promoters have also been demonstrated by ion treatment [7]. Reactivation of the mutant p53 with a new class of zinc metallochaperones (ZMCs), also by restoring normal structure and function to the zinc-free mutant p53, is made possible by the addition of zinc ions in extracellular environment [45,93,94,108]. For example, ZMC1 could stimulate apoptosis in p53 with *R175H* mutation and stimulate wt conformation change to the mutant protein [36,109]. Given the role of normal BRCA1 and p53 reactivation in tumor cells, it is hypothesized that both tumor suppressor proteins should be targeted by a novel biomolecule, such as zinc ZMCs [110]. This may be an effective treatment for BC because both proteins are in a malignant breast cell and are functionally inactive. In addition, the use of albumin nano vector (ANV) formulation has been suggested to release the target drug [110]. Research has shown that ZMC1 and its new drug formulation in complex with zinc ion could help tumor growth suppression and better survival, especially for the zinc deficient allele in BRCA1-deficient BC models [111]. In this research, the high synergistic influence of ZMC1 along with the Poly (ADP-ribose) polymerase (PARP) inhibitor olaparib has been observed. In addition, olaparib-resistant tumor cells are sensitive to ZMC1 treatment [111]. However, to investigate the safety and effectiveness of these approaches in clinical trials, more research is necessary.

### 9.2 Chaperone inhibitors

The reversibility of the structural changes of the mutant p53 is very important in that it is observed, especially in many temperature-sensitive mutants; thus, the activity of wt p53 is eliminated at 37 °C and regained at 32 °C [7]. In this regard, another study has been conducted on the effects of DnaJ Heat Shock Protein Family (DNAJA1) [also known as HSP40] molecule on the cells containing the mutant p53 [112]. This molecule, with its chaperone role, protects the mutant misfolded p53 against proteasome degradation [113]. Moreover, previous studies have proven that the degradation of the mutated p53 suppresses the tumor. Based on these observations, it was hypothesized that by inhibiting DNAJA1, the mutant misfolded p53 is degraded, which results in the inhibition of malignant features in cancer cells. For example, PLINH compounds, derived from plumbagin, hinder the growth and migration of cancer cells containing DNAJA1-mutant p53-dependent structural mutations [8,114]. However, DNAJA1 inhibitors are not yet available in either commercial or clinical settings, and additional research is necessary to examine the safety and effectiveness of these compounds as potential cancer treatments [8].

### 9.3 Small molecules and synthetic peptides

**Fig. 2:**
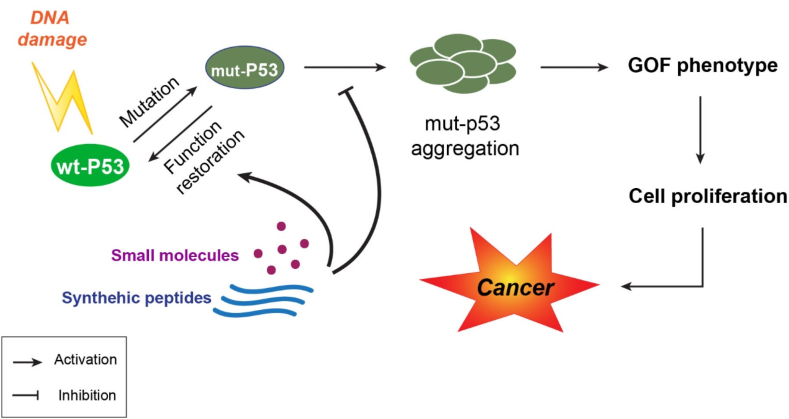
The suppressive role of small molecules and synthetic peptides in cancer development **Mut**: Mutation; **GOF**: Gain of function.

Various molecules, such as small natural or synthetic molecules, peptides, and nucleic acid aptamers inhibit prion-like behavior by stabilizing proteins and preventing the process of fibrillation and/or oligomerization and eliminating malignant behaviors [92,96,[115], [116], [117], [118]]. MDM2 suppresses the negative regulation of p53, which leads to multiple effective treatments. In cases of wt p53 expression, small molecules or peptides that interact directly or through secondary pathways with the p53-MDM2 can restore its tumor suppressor activities and prevent p53 degradation [20,119] (Fig. 2). As mentioned above, proper binding of p53 to DNA often decreases the formation of p53 aggregation. One study group reported that a double-stranded twin DNA stabilized the DBD domain of the p53 and inhibited amyloid formation [11,120].

#### 9.3.1 Small molecules

It has been revealed that CP31398, P53R3, PRIMA-1, and its methylated derivative, APR-246, can increase the stability of several mutations in the DBD domain, prevent wt and mutant p53 aggregation, and restore their tumor suppressor function and original conformation [29,42,[121], [122], [123], [124]]. APR-246 (Eprenetapopt) is the most investigated small molecule drug that targets cancer cells with mutated TP53 genes [125].

It is well-documented that both PRIMA-1 and APR-246 can stimulate apoptosis and reduce cell proliferation *in vitro* and *vivo* in various cancers, particularly in breast tumor cells containing mutant p53 [122,[126], [127], [128], [129]]. Noteworthy, APR-246 has more pro-apoptotic potency than PRIMA-1 and is known as the most investigated and advanced clinically mutant p53 reactivating compound [44,127,130]. APR-246 is a potential new treatment using apoptotic induction for p53-mutated BC, especially for TNBC subtype [19]. Assessment of the anti-cancer potential of APR-246 in combination with various clinically used chemotherapy agents has revealed that this compound along with eribulin has a high synergistic cell growth repression in several p53-mutated BC cell lines [131]; however, contrary to eribulin, the anti-proliferative influence of APR-246 in combination with doxorubicin, docetaxel, carboplatin, or cisplatin was related to cell line [19,131]. Immunoprecipitation, immunofluorescence, and size-exclusion chromatography showed the capacity of PRIMA-1 to impair mutant p53 aggregation in the BC cell line MDA-MB-231 containing R280K mutation. PRIMA-1 could also repress mutant p53 prion-like features *in vitro* [122]. Resembling APR-246, PK11007 can participate in apoptosis stimulation and suppress the growth and migration of mutant p53 BC cells compared with wt p53 BC cell lines. Similar to APR-246, it can enhance reactive oxygen species (ROS)production and restore the normal function of the mutant p53 in BC cells [[132], [133], [134]]. TNBC cell lines are more sensitive to PK11007 rather than non-TNBC cell lines [132].

COTI-2 (thiosemicarbazone) was determined *via* a dedicated computational platform called CHEMSAS®. It has been revealed that this compound has anti-cancer activity in the xenograft model of the MDA-MB-231 BC cell line and other cancer cell lines and xenograft models. Moreover, it can restore the wt p53 function and suppress the PI3K/AKT/mTOR signaling pathway [20,135].

Other p53 reactivating compounds, such as PhiKan083 and PhiKan7088, induce transcription of p53 gene targets, which can restore wt p53 folding, especially in the cells containing Y220C mutation in the DBD of p53 [136,137]. This process can terminate to cell cycle arrest *via* activation of p21 and Noxa-dependent apoptosis [[138], [139], [140]]. PhiKan7088 works synergistically with Nutlin-3a to further overexpress p21 and Noxa, which confirms the restored structure of mutant p53 [40]. Nutlin-3 has activity against a wide range of cancers containing wt p53 *in vitro* and *in vivo*. p53 transcriptional activity is promoted by it in osteosarcoma, retinoblastoma, colon, lymphoblastic leukemia, and BC cell lines [141,142]. Tonsing-Carter et al. applied a platinum-based regimen in combination with Nutlin-3a. A panel of TNBC cell lines with mutant p53 received both single and combination treatments of Nutlin-3a and carboplatin, and it was found that using Nutlin-3a could result in the activation of various anti-cancer pathways [143].

Furthermore, NSC319725 and NSC319726 are novel compounds that are known as re-activators of mutant p53 [144]. The metal-ion chelating features of NSC319726 can convert mutant p53 to change to a wt-like conformation. Indeed, NSC319726 activity was dependent on R175 mutant p53. These mutant proteins are unable to bind to zinc, but treatment with NSC319726 and ZnCl_2_in low concentrations can facilitate zinc binding [145]. In fact, variations in any zinc-coordinating residue can prevent zinc from binding to p53. However, the R175 mutation is not directly implicated in zinc binding; instead, a histidine residue in this area causes structural damage in p53, resulting in zinc binding inhibition [144].

Resveratrol (*trans*-3,4′,5-trihydroxystilbene), another molecule found in many plants, such as berries, peanuts, and grapes, has been reported to adjust numerous cellular targets involved in cancer signaling pathways and is able to inhibit aggregation of various amyloid proteins [[146], [147], [148]] including p53 in a BC model [148]. This study in 2018 revealed that resveratrol could inhibit aggregation of the DBD region through wt p53 and mutant p53 (R248Q) using a dose-dependent procedure *via* light scattering. Furthermore, in this experiment, resveratrol was tested in HCC-70 (R248Q) and MDA-MB-231 (R280K) mutant p53 BC cell lines, and these cell lines were more sensitive to resveratrol treatment rather than the wt p53 MCF-7 cell line [148]. Immunofluorescence experiments have shown that this treatment could decrease colocalization between p53 (DO-1) and amyloid oligomers (A11) in mutant p53 cell lines, and lower p53 aggregations were also detected in histological sections of MDA-MB-231 tumors in BalbC/Nude mice xenografted and they were treated with resveratrol [98,148].

Particularly, the development of small molecule modulators of the enzyme active sites of Inositol-requiring enzyme 1 (IRE1α) and PERK is of interest. A number of small molecules bind to and repress IRE1α′s RNase. A reactive electrophile that binds covalently to the RNase active site of IRE1 is present in most of the identified direct inhibitors [21]. These salicylaldehyde-based inhibitors generate a Schiffs base involving K907 (RNase domain) in the active site of RNase [149]. For instance, IRE1α RNase inhibitors have been shown to increase the response to chemotherapy and reduce the tumor cell secretome in xenograft models of TNBC [21,150].

#### 9.3.2 Synthetic peptides

A synthetic cell-penetrating peptide, RecACp53, can inhibit the accumulation of mutated amyloid p53 [98]. RecACp53 binds to the amyloidogenic portion (amino acids 252–258) of p53 (that is predicted by ZipperDB software) and prevents aggregation and mass formation *in vitro* [151,152]. RecACp53 can also reduce growth and metastasis *in vivo* and decrease the chemoresistance (e.g.*,* cisplatin treatment) in cancer cells [153]. Interestingly, this molecule has no effect on the cells containing wt p53 [11,151]. Although by targeting mutated p53 and preventing the formation of amyloid fibrils and clumps, these methods have given rise to bright hopes for the early detection and prevention of cancer, unexpected side effects can result from therapies that target a specific protein or intermolecular interaction [11].

Furthermore, it has been demonstrated that in a BC cell model comprising full-length R175H mutant p53 (i.e., SK-BR-3 cells) polyarginine can restore wt p53 activities. The enhancement in p21 expression levels after treatment with polyarginine, detected *via* immunofluorescence, is suggested to be a demonstration of p53 reactivation [98,154]. However, further experiments are needed to assess the correlation between the enhancement of p21 expression and impairment of p53 aggregation [98].

Another peptide, which can target both MDM2 and MDMX, is ALRN-6924 [155]. Although it has high efficiency against different BC cell lines harboring wt p53, cells containing mutant p53 were persistent [156]. Additional investigation is required to examine the safety and efficacy of these molecules and peptides as potential cancer therapies.

### 9.4 Aggrephagy-based approaches

**Fig. 3:**
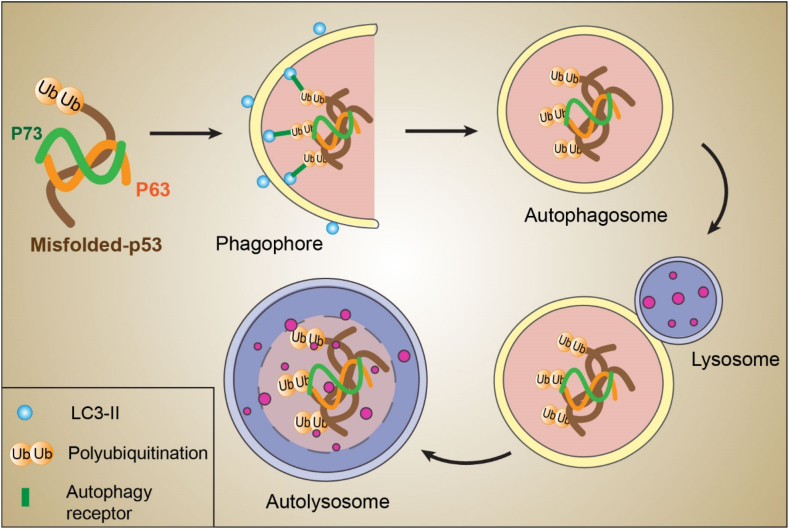
The aggresomes of misfolded p53, p63, and p73 are recognized by autophagy receptors and then are directed to autophagic degradation. The process of autophagy includes the formation and elongation of the phagophore membrane; phagophore membrane swallows the protein aggregates to create autophagosomes. Fusion of the autophagosome with lysosomes leads to create autophagolysosome that protein aggregates degrade *via* lysosomal proteolytic enzymes. **LC3**: Microtubule-associated protein 1A/1B-light chain 3; **UB**: Ubiquitin.

Mutant p53 aggregation with mutant/wt p53, p63, and p73 can form large inclusion bodies, which are unable to enter the proteasome; thus, they cannot be degraded, which ultimately leads to increased concentration in the intracellular environment [157]. These cytoplasmic inclusion bodies, which are ultimately degraded by autophagy, are known as aggresomes [158]. The decomposition of mutant p53 aggresomes with misfolded protein aggregates through specific autophagy is called aggrephagy [159], and this phenomenon is described in detail in Fig. 3.

The assessment of functional autophagy with damaged aggrephagy was performed in 2020 on an experimental model with mitophagy (i.e., mitochondrial dysfunction) in BC cells (MDA-MB-231) containing R280K mutant p53 with defective degradation of p53 aggregates [160]. The results indicated the autophagy receptors nuclear dot protein 52 (NDP52) and TAX1 BINDING PROTEIN 1 (TAX1BP1) were fragmentized through autophagy (i.e., functional autophagy) even when p53 aggregates accumulated (i.e., aggrephagy repression). They indicated these autophagy biomarkers are suitable for monitoring autophagy and can be used in personalized medicine for patients with mutant p53 [160]. Another study in 2016, based on a dietary approach, revealed that BC cell line SK-BR-3 was empty of R175H mutant p53 aggregates using phenethyl isothiocyanate (PEITC) extracted from cruciferous vegetables. PEITC can restore wt p53 tumor-suppressive activity to mutant p53 that eventually terminates to more sensitivity to MDM2-dependent proteasome degradation [161]. To examine the efficacy and safety of aggrephagy-based approaches in treating cancer, more research is needed [160].

### 9.5 P53-based immunotherapy approaches

**Fig. 4:**
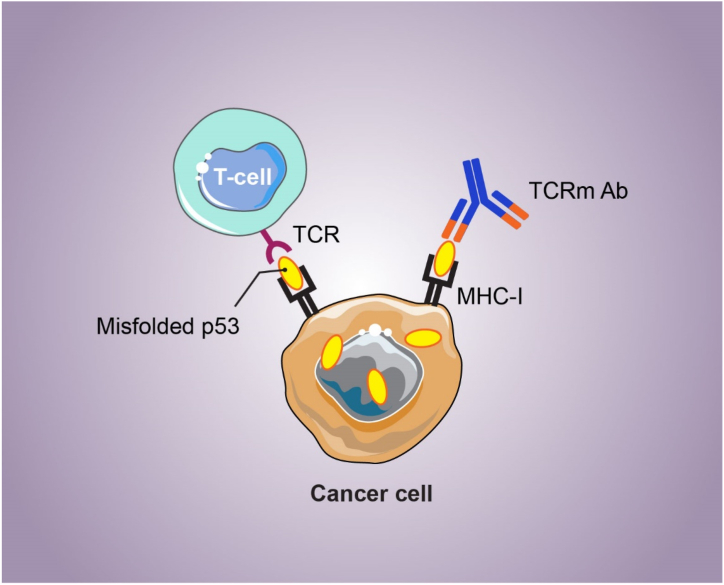
Schematic view of p53-based immunotherapy using TCRm **TCR**: T-cell receptor; **TCRm**: T-cell receptor mimic; **Ab**: Antibody; **MHC-I**: Major histocompatibility complex class I.

One of the p53-based immunotherapy approaches is the use of T cell receptor mimic (TCRm) antibodies (as well as known as TCR-like antibodies), which are a potential procedure to target intracellular proteins [162]. Hybridoma screening or phage display library screening produces antibodies that can recognize epitopes exposed by major histocompatibility complex (MHC) class I on the cell surface, or recognize them *via* TCR of T cells [163] (Fig. 4). Accordingly, a recent TCRm antibody was developed that identified a p53-derived epitope which is selectively exposed on MHC class I *via* cancer cells. Mice with BC xenografts exhibited tumor regression in response to this p53 TCRm antibody [164].

### 9.6 P53-based gene therapy approaches

Gene therapy methods totally act *via* various mechanisms, such as gene knockdown, insert a new gene, replacing malfunction genes with the therapeutic genes, and deactivating problematic genes for disease treatment [165]. Gene therapy approaches are able to treat various diseases including cancers efficiently owing to elevated comprehension of the pathogenesis of disease and improved gene delivery methods [166]. P53 gene therapy is made more impressive and feasible by the use of complicated viral vectors, especially as part of combination regimen treatments [167]. Also, nanoparticles have been investigated as vehicles for p53 gene therapy and their low immunogenicity makes them resistant to inhibitory antibodies (contrary to viruses); thus, their circulation time can increase and at the same time the side effects related to the immune system are reduced [163]. Moreover, these vehicles are more adequate for remedying distant metastases using intravenous injection versus intratumoral injection. Importantly, improved methods for delivering gene products, especially for cancer treatment, have significantly enhanced the specificity and efficacy of nanoparticles, increasing their potency to restore the expression of p53 selectively in cancer cells and exert stronger anti-cancer outcomes *in vitro* and in numerous xenograft models, including those of hepatocellular carcinoma (HCC) and BC [168,169].

#### 9.6.1 Small interfering RNA (siRNA)

Synthetic siRNA delivery can be used in p53-targeted genetic therapy to target specific mutations in p53 mRNA and suppress the GOF effects of mutant p53 [163]. A single base difference in the siRNA has been demonstrated to be enough to distinguish between mutant and wt p53 [170]. Currently, Ubby et al. have invented specific siRNAs for four various hotspot mutations in p53 and indicated that they were capable of reducing the livability of patient-derived xenografts in a mutant-specific method with no effect on wt p53 mRNA without organ toxicity [171]. Since p53 can elevate the convergence of the intrinsic and extrinsic apoptotic pathways, such as in TNBC cells, a study has indicated that suppressing the overexpression of mutant p53 in TNBC cell line (Hs578T) can lead to the activation of pro-apoptotic genes including inflammatory caspases, BCL-2 gene family, and death receptors and downregulation of anti-appoptotic gene BNIP2, which ultimately promotes paracrine and autocrine cell-mediated death [172]. Despite the infancy of siRNA-based therapies, these findings and the fast advancement of RNA delivery technology may drive more research into mutant-specific p53 siRNAs for cancer therapy [163].

#### 9.6.2 Clustered regularly interspaced short palindromic repeats (CRISPR) technology

Clustered regularly interspaced short palindromic repeats/CRISPR-associated protein 9 (CRISPR/Cas9) is a powerful method for the investigation and treatment of different cancers, such as BCs [173]. For instance, the correction of 414delC null mutation of TP53 in PC-3 prostate cancer cells using CRISPR terminated enhanced protein expression and apoptosis, highlighting its capability for correcting mutations of this gene in BC [174]. Moreover, in research introducing CRISPR-Cas9 base editing (Cas9 conjugated to adenosine or cytidine deaminases), which provides improved specificity and fewer indels (insertion/deletion), the base editor was used to turn a missense mutation (Tyr163Cys) in TP53 in HCC1954 BC cells into a wt sequence [175]. Importantly, in cells that contain wt p53, CRISPR-Cas9 stimulates p53 activation after DNA damage, which results in cell death or cell cycle arrest; thus, this is a selective approach used to survive cells with TP53 mutations [176]. Similarly, cancer cells harboring mutant TP53, in which conversion into wt TP53 is achieved successfully, might be removed more effectively because of simultaneous DNA damage signaling. However, successful conversion must be obtained from a wide variety of cancer cells for clinical benefits [163].

Noteworthy, there are serious doubts about the effectiveness of such approaches, particularly if p53 reconstitution is not combined with other therapeutic aims. For example, although degradation of p53 is a hallmark of HPV^+^ cancer [177], it seems that enhancing p53 expression (using CRISPR) alone is not enough to prevail this and might need a combined method, such as conventional chemotherapy [178,179]; because despite p53 initial hyper-expression, which can terminate to tumor death, the generation of prion aggregations may cause a conflicting stimulation of another cancer [180]. In addition, the rate of success of gendicine (i.e., a drug based on gene therapy that is being used to treat different types of cancer) and its proper application in cancer treatment is still being debated. Thus, further research is required to investigate the safety and effectiveness of these p53-based approaches, particularly if combined with other therapeutic targets, and can help to develop personalized medicine approaches for cancer patients [163].

## 10 Conclusion

Understanding the underlying molecular mechanisms of BC pathogenesis is crucial for developing more effective prevention and treatment strategies. Mutant p53 proteins can aggregate and form prion-like structures, which may contribute to the pathogenesis of BC. The Prion-like behavior of carcinogenic p53 mutations explains their DN and GOF characteristics; specifically, it can be considered as having a high potential for metastasis in cancer cells carrying the p53 mutation. Our objective in this review is to provide an overview of the strategies used to inhibit p53 aggregation and degradation, which are promising targets for cancer therapy, particularly in TNBC, which is a high-prevalence BC in young women with invasive features. Thus, this article provides an extensive review of various approaches to targeting mutant p53 aggregates and highlights the need for continued research to explore their potential safety and efficacy in clinical trials.

## Founding

None.

## Data availability statement

All data generated or analysed during this study are included in this published article.

## Additional information

No additional information is available for this paper.

## Ethical approval and consent to participate

Not applicable.

## Consent for publication

Not applicable.

## CRediT authorship contribution statement

**Yasaman Naeimzadeh:** Writing – original draft, Visualization, Validation, Methodology, Investigation, Conceptualization. **Amir Tajbakhsh:** Writing – review & editing, Writing – original draft, Supervision, Project administration, Investigation, Formal analysis. **Jafar Fallahi:** Writing – review & editing, Validation, Supervision, Methodology, Conceptualization.

## Declaration of competing interest

There is no conflict of interest.
